# The impact of the narrative mindset on effectivity in social problem solving

**DOI:** 10.1371/journal.pone.0253729

**Published:** 2021-07-01

**Authors:** Jerzy Trzebiński, Jolanta Zuzanna Czarnecka, Maciej Cabański

**Affiliations:** Department of Psychology, SWPS University of Social Sciences and Humanities, Warsaw, Mazowsze, Poland; University of St Andrews, UNITED KINGDOM

## Abstract

The narrative mindset is a tendency to interpret social information in the frame of stories. Two experiments were conducted to determine if and why the narrative mindset increases social problem-solving effectivity. The experiments consisted of two parts: the experimental manipulation (inducing the narrative mindset or control condition) and the observation of effects. In the second part, presented as a separate study, a participant was asked to advise other people facing interpersonal problems (experiment 1) or emotional problems (experiment 2). Three pairs of coders judged each piece of advice independently on three scales: Effectivity of the advice, empathy, and personalization (using their own experiences in providing the advice). The results indicate that the narrative mindset increases empathy, supported by the co-occurring increase in the problem’s personalization, which leads to higher effectivity. The results reveal the positive real-life implications of structuring social information within a story frame. It may encourage the introduction of the narrative mindset effects into an area of social cognition research. Finally, the experiments show that the narrative mindset may be activated experimentally, providing an effective instrument to test the impact of narrative knowledge on social cognition.

## Introduction

Our study inquires if and why the narrative mindset increases effectivity in social problem-solving. In a broader sense, social problem-solving means a mental process to find an effective solution for problems (not necessarily strictly social) encountered by ordinary people in everyday living [1]. Of the different types of social problems that people face, we addressed two dominant ones: interpersonal, in which difficulty arises in dealing with others, and emotional, where the point of focus is an internal struggle with an emotional state [1–5].

Studies on social problem-solving concern mainly different negative correlates of effectiveness in solving, such as depression [3, 6–10], schizophrenia [5, 11], previous suicide attempts [12], lower social adjustment [2, 13], older age [14–18], weakened episodic memory recall [14, 16], and physical, neurophysiological, and psychiatric impairments and deficiencies such as amnestic mild cognitive impairments [19] and temporal lobe epilepsy [18].

Up to date exploration of situational factors, including mindset, influencing the ability to solve social problems has been relatively scarce. Factors tested include negative mood induction [20], a sequence of strategies to deal with negative affect [21], and preceding problem solving with laboratory tasks on episodic memory elaboration specificity [8, 10, 14, 15].

In two experiments, we tried to observe the impact of the important and widespread narrative mindset on the effectiveness of solving interpersonal and emotional problems. Previously reported properties of the narrative mindset encouraged us to verify its likely influence on improving problem-solving effectivity.

### Theoretical background: Narrative mindset and narrative knowledge

The narrative mindset is a tendency to interpret social information in the frame of stories. Stories are an essential tool for structuring and comprehending social behavior and a person’s experiences [22–28]. The building blocks of stories are motives, and the complications that induce, impede or block their fulfillment. In construing understanding of events and situations as stories, or people as stories’ protagonists, an individual uses general knowledge about human motives and specific contexts of evoking and pursuing them [29–33]. This narrative knowledge is developed during social interactions [34, 35] and is influenced by culture. At least in contemporary societies, it is specific for different social domains [36–38]. An example is the self-story: a stabilized knowledge structure that influences a person’s identification and decisions [39–43]. In most parts, it is shared with others (among members of one’s group or society).

When the narrative mindset is activated, the narrative knowledge is ready to understand and memorize incoming social information. As a result, a narrative mindset tends to structurize social information in story plots: the protagonists with intentions, obstacles, conditions of fulfillment, and timely ordered routes of striving for them [44–47]. There are several other influential and common tendencies in processing social realities. Some of these non-narrative processes are called paradigmatic [23–48]. The diverse examples for this kind of information processing include comparing objects, traits, and psychological states, trait attribution in person perception, social categorization, stereotyping, scientific explanations, creating and applying the law and regulations, and learning knowledge in school. However, it seems that the narrative mindset and knowledge greatly influence our everyday social interactions [26].

### The empirical background: Impact of the narrative mindset on social cognition

Narrative knowledge and mindset result in understanding events as stories, but it is also a basis for other kinds of social cognitions [49]. The narrative mindset is likely to be a very human frame for making an impression of another, understanding their internal states, motivations, problems, and creating possible future simulations or finding solutions for social problems.

The basic building blocks of the narrative mindset and knowledge are intention categories like wishes, goals, plans, and emotions. Goal priming experiments provide evidence of the narrative mindset activating the intention category. If goals or motives are a major mental category of narratives, a narrative mindset should be a potent means of goal priming. Evidence shows that once primed, goals are accessible and have the potential to influence behavior for some time [e.g., 50, 51], but once a goal is fulfilled, goal-related information is deactivated, becoming less accessible and less likely to influence subsequent behavior [e.g., 52, 53]. Laham and Kashima [54] used this observation to distinguish the priming of a goal as a mental category from simple semantic priming by the goal’s term. Participants were primed with high achievement either after the narrative mindset activation or in a non-narrative context. They then completed either a behavioral task, followed by a measure of construct accessibility, or a behavioral task after a delay. Indicative of goal category priming, narrative-set participants showed more significant post-delay behavioral assimilation and less post-fulfillment accessibility than those exposed to the non-narrative context.

The intention category is a key factor in structuring social reality as meaningful stories and comprehensive events. The crucial role of the intention category in processing incoming information is evidenced by observations from cognitive psychology and psycholinguistics [55]. For example, being unable to discern the intention behind a segment of actions disturbs recognizing these actions as a single event [56, 57]. People spontaneously impose default motives when detecting complex facts and actions as a story or an understandable event [58]. Furthermore, different information regarding motive or emotion affects observers’ interpretations of the same chain of behaviors. Jury transcripts provide naturalistic observation. It appears that hypotheses for the defendant’s motive determine what evidence the jury is looking at and how it is interpreted, which subsequently influences the defendant’s conviction of guilty or innocent [59, 60].

The above data indicate that understanding in a story form means knowing the intention and applying this knowledge in processing information. The narrative mindset’s priming should increase the significance of the intention and emotion’s mental categories in understanding another person [61]. We assumed that it effects positively the quality of person impression. In one experiment, participants received a description of a young person presented with a professional label, e.g., "a policeman" [62]. They were asked to freely describe their impressions of the person and choose from the provided list a specified number of words that characterize the person best. Before the presentation, they participated in the narrative priming (writing a story based on provided pictures) or one of two neutral conditions (grouping social objects, no activity). After narrative priming, the impressions were scored by independent coders as more psychologically coherent, easier to imagine, and more complex compared to the neutral conditions. Furthermore, their authors were more confident in the validity of the impressions and the distinctness of the person. The narrative primed participants chose more words related to the motive domain as best descriptions of the person’s character and fewer words stereotyping the person e.g., specific to the professional category. In another study, participants obtained a person’s description that was divided into two sets, portraying two different personality profiles, and were asked to describe their impression of the person. Independent coders evaluated the descriptions after the narrative priming as more effectively integrating the inconsistent characteristics [63]. In conclusion, it seems that–compared to other everyday contexts of person perception–the narrative mindset facilitates impressions organized around a person’s motives and emotions. These are more individualized and less stereotyped, and easier to visualize than in other person perception contexts. These impressions are more coherent psychologically, even when based on inconsistent data.

The following studies observed the impact of the narrative mindset on the readiness to evoke motive-related categories in an impression of a person, measured by reaction time [62]. The attribution was in the form of fast *yes* vs. *no* decisions to state whether the person may possess a specified characteristic from a provided list. Before presenting the psychological portrait of the person, the participants were primed by a task inducing the narrative mindset, a neutral task, or were not primed. Provided characteristics belonged to general personality traits (*quiet*, *straight*, *pessimistic*) or broad motives (*loves*, *strives for success*, *jealousy*). It appeared that the narrative priming shortens the decision reaction time case of motive attributions, but not trait attributions. This effect was observed at the level of differences in fast reactions, which are not controlled consciously. It allows a conclusion that the impact of the narrative mindset and activated narrative knowledge on social cognition is independent of the level of conscious and effortful control, which is typical for schema-driven mental processes. When activated, e.g., by priming, they regulate conscious thinking [63].

We further expected that, because the narrative mindset accentuates the motives and emotions and provides a framework to integrate them into a meaningful vision of a person, this mindset may also induce a better understanding of a person in their specific situation, for example, in difficulty. It may take the form of empathizing with a person in need and may lead to helping. It should be easier to understand and feel a person’s problem or tragic situation, and subsequently help them if we know this person’s story, even if it is only indirect. Mass media provide a lot of examples. TV news about a critical drought and hunger in Ethiopia in 1991 illustrated by one victim’s life story, a small girl from an Ethiopian village, lead to higher financial donations than presenting some tragic statistics [30].

In one experiment, participants were informed they would be participating in an informal meeting organized by the medical service in which they would be asked to consider helping individuals with leukemia [64]. Each subject was randomly given one of two texts: a description of the life of an ill man with leukemia or information about leukemia and the role of bone marrow transplantation in therapy. Next, they were asked to decide whether they wanted to participate in a bone marrow donation program. Finally, the participants filled out a test measuring their general ability to detect the narrative structure in provided material [65]. The data confirmed that the presentation of a story of an ill person related to a higher percentage of declarations of help (which was verified after two days). Still, this effect occurred almost exclusively among those who had a higher narrative competency. It may suggest that the observed effect’s main factor was the narrative mindset activated more easily in those who could extract and understand the narrative message of the text in a more elaborate way. We observed from the literature that reading a story about a person, or category of persons in need, increases the probability of helping. Narrative priming before the presentation of a person in need strengthens the willingness to spend time on the phone soliciting money for an ill person and readiness to take a course preparing to be a member of the Big Sisters–Big Brothers program [62].

We may conclude that being in the narrative mindset evokes a more straightforward empathic understanding of a person in need. Activated narrative knowledge provides a framework that enables identifying and integrating a person’s motives, emotions, and situational context in a coherent vision. It attracts attention, arouses feelings and moral convictions, and increases the willingness to help.

We supposed that there is still another kind of narrative mindset that influences understanding of other people’s circumstances. The narrative framework may facilitate imagining oneself in the other person’s situation [66]. We called it the personalization of other people’s problems. This facilitation results from structural similarities between understanding other people’s problems within a story frame and knowledge emerging from a person’s life experiences–their experiential knowledge. Experiential knowledge is organized in episodic memories and scripts based on repetitive social events [30]. This knowledge is transformed creatively and is enriched by memories shared with close ones. [67, 68]. It is hypothesized to have a narrative-level organization proven by a better recall of events that form coherent narratives than ones that do not [69]. Linguistic analyses of protocols with spontaneous descriptions of memories collected in different life situations indicate that experiential knowledge is essentially about trajectories of motivated actions and related emotions modified, blocked, or helped by incoming situations [e.g., 70–72]. When the narrative mindset facilitates story framing of other people’s difficulties, thinking about someone else’s difficulty activates their experiential knowledge more effortless because of that similarity. We posit that personalization of someone else’s problem is especially useful in situations with no ready-to-use advice, such as common-sense advice learned and socially shared as behavioral scripts or proverbs [73]. This "(…) forces the problem-solver to abstract elements from his own subjective experience and to utilize these elements as the foundation for highly subjective solutions" [5, p. 232].

Our experiential knowledge plays a significant role in reasoning, such as social problem-solving and imagining and planning the future [74]. Neurological studies show that damage to brain areas supporting episodic memory coincides with a lowered ability to imagine future and possible events [75, 76]. Episodic memory processes, mediated by the medial temporal lobes, contribute to open-ended problem-solving [18]. Furthermore, after a brief training session for recollecting details of past experiences, young and older adults perform better on memory and imagination tasks, as well as means-end problem-solving tasks [15, 77]. On the other hand, lowered autobiographical memory after posttraumatic stress disorder relates to ineffective social problem solving [78]. Parasuicide patients tend to retrieve overgeneral autobiographical memories (i.e., with lesser specific emotional content), which relates to problem-solving deficits [79, see also: 80]. High alexithymia (inability to verbalize emotions) correlates with deficits in both episodic memory [81] and mentalizing skills, such as perspective-taking mental imagery, fantasizing, and narrative transportation [82–84].

In conclusion, experiential knowledge is a helpful resource in thinking, especially in the social domains. Structural similarities between the narrative understanding of a person coping with a problem and our personal experiences will spontaneously prompt us to wonder how we would feel and cope in the same situation, making it easier to find a solution. We expected that activating personal experiential knowledge would result in a more elaborate understanding of the problem and, therefore, more effective thinking about solving it.

### The hypotheses

Based on the presented data and its interpretations, we expected that the narrative mindset facilitates an empathic understanding of someone experiencing trouble and seeking a solution. The activated narrative knowledge enables one to better understand and efficiently think about another person’s intentions, emotions, and situational context. The narrative interpretation of the problem also opens a way to personalize it: to use personal experiences to understand the dilemma and offer good advice.

In two experiments, we verified the connected hypotheses that evoked narrative mindset facilitates (1) empathic understanding of another person with a problem, (2) personalization of the problem, and subsequently (3) increases the solution’s effectiveness in the advice form. Effective advice is advice that (probably) resolves the problem when applied. In our research, the measure of advice’s effectiveness was based on the coders’ evaluation of the extent to which the advised action may solve a given problem in reality. To broaden the verification data, we observed the impact of the activated narrative mindset in two cases of social problems often analyzed in classical research: interpersonal (connected with other people) and emotional (struggle with emotions) [2, 5].

### Experimental activation of the narrative mindset

To observe the effects of the narrative mindset, we used an experimental procedure of mindset activation. Diversified data illustrate how easily simple word stimuli used as primes can activate a mindset that regulates thinking and behavior in a subsequent situation. Such word stimuli addressed a complex knowledge structure (e.g., the word "*mother*"). It was shown that using even simple words as primes (like *mother* vs. *colleague* or *friend* vs. *co-worker*) unconsciously activate different knowledge mindsets that provide criteria of person evaluation, affect the decision to help, stipulate specific goal-oriented behavior or behavioral stereotypes, or affect the likelihood of aggressive or polite behavior [52, 85–88].

The narrative mindset is frequent and influential among people. It may be induced when listening, reading, or watching pictures or movies and making a story [89]. In our study, the narrative mindset was activated by a task to create a story illustrated in the provided pictures. Providing a task to perform specified action (hammer a nail) and then observing transitory readiness to use the same action to cope with a different task (repairing a watch) is classical experimentation on a mindset effects on problem-solving [90, 91].

There are three benefits of using such a procedure. First, the task used to evoke the mindset, like "create a story illustrated in provided pictures", is a simple instruction, understood equally by all participants. Similarly, the stimuli–pictures in this case–are easily understood. Finally, the control condition might be closer to fulfilling "the smallest difference" requirement as participants are working on the same set of pictures as the narrative set participants, performing a known ordinary task of grouping objects according to their similarity.

As in many studies on social problem solving, a participant was asked to provide a solution to a problem presented in a 3^rd^ person form, meaning it was a solution for the other person, not their own problem [92]. This may eliminate the influence of different uncontrolled personal factors. We took an open approach in observing the impact: the participants were presented with a problem and had freedom to invent the best advice. This allows better inquiring about the expected effects of the narrative mindset on the advice’s content characteristics.

## Method

### Ethics statement

The studies involving human participants were reviewed and approved by Research Ethics Committee at the SWPS University, Department of Psychology. The patients/participants provided their written informed consent to participate in this study.

### Plan of the two experiments

Two experiments had the same structures and similar tasks for the participants. They differed in the kinds of problems to be solved. There were interpersonal problems in experiment 1 and emotional problems in experiment 2. The experiments consisted of two parts. In the first part, all participants were assigned randomly to one of two conditions: inducement of the narrative mindset or the control condition.

**In the narrative mindset condition,** each participant was presented with six pictures depicting expressive scenes with people interacting. The same woman and man appeared in each picture, together or alone, and with other people in one of the pictures. A participant was asked to look at the pictures and imagine a story that might be illustrated on them and then describe the story: "*Describe the story that you thought about when looking at the pictures*. *Describe how the story started*, *what is happening in it*, *and how it ends*. *Describe what the characters are all about and what is bothering them*, *and why all of this is important for them*. *Do not restrain yourself in any way when searching for a meaningful story*".

**In the control condition**, each participant obtained the same array of pictures as used in the narrative mindset condition and was asked to categorize them into all subcategories that come to mind that have a common feature and then to describe each feature: "*Your task will be to find such sets of pictures that have a common unique feature*, *a feature that the rest of pictures don’t have*. *You can take various things into consideration*, *even unobvious ones*: *the type of place*, *the accessories*, *the way the people appear or behave*, *their emotions*. *It could be the way they are dressed*. *You can take into account the wider environment in which the pictured places might be*. *Do not restrain yourself in any way when searching for the common features*".

Participants could take as much time as they needed to complete the task. In the pilot studies, no significant differences were observed between the two experimental conditions on scales of instruction understandability or task difficulty, or task completion.

At the end of this part, the participants were told this was the end of study one and were invited to participate in study two (which was the second part of the same experiment). The second part of the experiment was a social problem-solving task. In experiment 1, participants were asked to provide advice on how to solve four interpersonal problems, and in experiment 2, on how to solve four emotional problems. In each case, the problem’s description included brief information specifying a particular person’s needs, like some characteristics of the person and the social environment. Participants’ task was to invent the best solution in the form of advice. Therefore, it was a divergent version of the problem-solving procedure: a participant was free to create the solution. In other studies, this procedure is usually more convergent: a participant is provided with the need and positive ending and is asked to find steps leading to such an end [4]. We assumed that the divergent version would facilitate a more elaborate search for the expected mindset-related differences in the advice content.

In both experiments, the sample sizes were determined following previous studies on social problem solving with experimental manipulation of episodic memory specificity induction [14, 15] and on the influence of narrative mindset on various social cognition tasks [62, 63].

### An objective analysis of the attentional focus of advice-givers

To provide a further assessment of problem personalization, independent from coders’ evaluations, we performed an analysis using the Linguistic Inquiry and Word Count (LIWC) 2001 program [93]. It analyzes texts by scanning them in a word-by-word manner and calculating words within specified categories. Verb tense and personal pronouns are indicators of a person’s focus. We believe that the number of *1*^*st*^ *person singular pronouns* (like I, my, me, myself) and *past tense verbs* (like walked, were, had) would be good metrics for the extent to which advice-givers utilized personal experiences [94, 95]. The use of *1*^*st*^ *person singular pronouns* is characteristic for situations when a person is focused on oneself and takes personal ownership of the reported experience [95, 96]. Personal pronouns and past tense verbs are characteristic of informal communication with colleagues or close ones, based on sharing personal experiences. Simultaneously, they are infrequent in complex and analytical thinking used in collaborative work on shared tasks. Next, past tense verbs are not common in communication with strangers that lack disclosure [94]. Texts describing personal stories can be differentiated from the other types of social media posts based on a higher frequency of past tense verbs and first-person pronouns [97]. The higher number of them, the closer psychologically the person is to the described events [98]. We expected that significantly more *1*^*st*^ *person singular pronouns* and *past tense verbs* would be present in the advice written by narrative mindset condition participants. However, based on previous studies using the LIWC [95], we supposed that correlations would be small to moderate rather than strong.

## Experiment 1: Interpersonal problem-solving

### Participants

The experiment finally included 109 mTurk users (70 men and 39 women) who correctly followed the instructions. Before analysis, 12 participants (7 from the experimental condition) were excluded by independent coders for one or two of these reasons: (a) possible cheating (the same text in more than one participant), (b) provided text was irrelevant to the task and was probably copied from other sources. Participants were recruited from the English language pool of the mTurk workers and paid $3.5 for their input. The experiment was conducted online and was done individually. Table 1 presents detailed demographics.

**Table 1:** Demographics of the participants in the interpersonal problem-solving study.

	*n*	*%*	*M*	*SD*
Age				
Total sample	109		35.04	10.51
Female	39	35.8%	35.08	10.63
Male	70	64.2%	35.01	10.52

### Procedure and materials

Participants were asked demographic questions (gender, age) and then randomly assigned to the narrative mindset activation (*N* = 53) or a control condition (*N* = 56). After fulfilling the first part (a task with the pictures), they were invited to the second one. Next, they received the following instruction: "*You will be presented with a description of four persons*, *each of them having specific problems to overcome*. *Your task is to invent a solution to each problem*. *Imagine that you give advice to each person*. *What would you advise them to do*? *Consider each problem carefully and then provide the best solution*. *Try to be detailed in the description of this solution*. *Imagine that each person has the same gender as you"*.

Then four problems from four different people were presented. Descriptions of problems were based on the MEPS task [4]. Descriptions were gender-neutral.

Four interpersonal problems were as follows:

"*A realizes that a friend is avoiding her/him*. *A wanted very much to make up with him/her*.
*B had just moved in that day and didn’t know anyone*. *B wanted to have friends in the neighborhood*.
*C must prepare materials for a presentation in 2 weeks together with a shy classmate with whom C has rarely communicated*.
*D was listening to people speaking at a meeting about how to make things better in her/his neighborhood*. *D wanted to say something important and to have a chance to be a leader too*."

The problem’s description ended with the task: "*What is your advice for [letter pointing on a person from task]*?". Participants were given an unlimited amount of time to describe their advice.

### Advice evaluation–coders’ assessment

Three pairs of coders, all of whom were postgraduate clinical psychology students experienced with elementary psychological training and advising in everyday interpersonal and emotional problems, worked independently on the provided texts with advice. They assessed the advice on three scales (each pair working on one scale):

*The scale of effectivity*–evaluates how well given advice would solve the person’s problem if applied. Coders were asked to assess the extent to which an application of given advice would solve the problem on a 5-points scale ranging from "1"–not solves to "5"–solves completely. They were instructed to consider the specificity of the person’s problem.

*The scale of empathy*–evaluates how deeply the provided advice is based on an empathetic understanding of the person in need. Coders were instructed to assess the extent to which participants referred to the person’s feelings, emotions, motives, and specific way of understanding and thinking (current or possible at the course of getting to a solution). The 5-point scale ranged from "1"–absent to "5"–very highly.

*The scale of personalization*–evaluates the degree to which participants introduced their own experience when formulating the advice. Coders evaluated how often a participant uses personal references such as recalling personal experiences and stating opinions and beliefs that stem from life experiences, both their own and those of friends or family. They might be explicitly used as an argument for the provided advice or read between the lines. The 5-point scale ranges from "1"–absent to "5"–very highly.

Before the scoring, coders were trained on material from the pilot studies. They were given detailed instructions and example pieces of advice written by participants of the pilot studies. Their first assessments were a topic of shared discussion to ensure their full understanding of the scales’ character and to calibrate the scales’ evaluation in a coordinated way. After that, coders worked independently.

Two coefficients were used to assess the inter-rater reliability, in line with previous studies that applied the qualitative scale assessment of problem-solving results [e.g., 9, 92, 99]. Pearson’s correlation coefficient was used to estimate the degree of agreement on the coders’ overall evaluations. These were the means of the ratings for the four pieces of advice which were calculated separately for each of the three dimensions. Coders’ evaluations correlated as follows: efficiency scale *r* = .89 (*p* < .001); empathy scale, *r* = .92 (*p* < .001), and personalization scale *r* = .87 (*p* < .001). The range of the correlations is similar to those reported in previous studies [e.g., 92].

The weighted kappa coefficient was used to assess the level of agreement on each piece of advice separately. This allowed for a more detailed evaluation of the inter-rater reliability. We chose the weighted kappa because it computes the adjacent ratings as partial agreements; thus it provides more accurate estimates for ordinal variables than the standard Cohen’s kappa [100]. We chose the linear weighting because it gives more reliable estimates when there is no reason to assume that the adjacent categories’ differences are of different magnitude [101]. The results can be found in Table 2.

**Table 2:** Interrater reliability of the coders’ evaluations of the pieces of advice—linearly weighted kappa (the interpersonal problem-solving study).

	Scale		
Advice	Effectiveness of advice	Empathy	Personalization of problem
1	.72	.74	.78
2	.57	.53	.66
3	.63	.71	.32
4	.72	.55	.62

In line with the Cohen’s guidelines, the level of agreement between the coders for the majority of evaluations can be judged as substantial (.61 - .80), in three cases as moderate (.41 - .60) and in the case of the third advice on the personalization of problem scale, as fair (.21 - .40). The final measures used in the statistical analysis were computed by calculating the average of the four ratings for each of the two coders in a given pair separately and then taking the mean of the two resulting values.

Examples of pieces of advice that illustrate high and low results on each scale are presented in Table 3.

**Table 3:** Examples from participants’ pieces of advice from both conducted experiments.

	Experiment 1: Interpersonal problem solving	Experiment 2: Emotional problem solving
***Scale of personalization***		
**Problem to solve**	D was listening to people speaking at a meeting about how to make things better in her/his neighbourhood. D wanted to say something important and to have a chance to be a leader too.	The husband/wife of B died five years ago in a car accident. Although a lot of time has passed, B is depressed. Almost nothing can put him/her in a good mood, s/he does not see much sense in anything s/he does. B would like to feel like s/he used to.
Low score	Breath, raise your hand, speak loud and slowly enough to be understood by others, smile, do your best.	My advise to B is to join a support group. Doing this will enable B to realize that others are experiencing similar losses and she can share her own feelings knowing that others are there to listen. She will also meet new people and gradually may come to start enjoying things more little by little.
High score	Speak up if he has something valuable to say, if not its just as important to be a follower. people wanting to be leaders for the sake of it, is not helpful.	When I was separated and then divorced I became incredibly depressed. There wasn’t anything joyful in the world for me. I alienated friends and family, I was lonely but still pushed people away. We can help each other. Let’s start with a walk in the neighborhood. Moving makes me feel better. We can chat as we go. I have seen a good counselor that you may like but let’s just start with a walk.
***Scale of empathy***		
**Problem to solve**	C must prepare materials for a presentation in 2 weeks together with a shy classmate with whom C has rarely communicated.	D often explodes with anger and becomes aggressive towards others, even towards loved ones. S/he has the impression that people are starting to avoid him/her. D feels that s/he cannot control his/her bad emotions.
Low score	I would tell C that the best they can do is sit down with their shy partner and see if they will be able to help with the verbal presentation. If they can’t, then having them work more on the building of the presentation might be a way that they can still help the team.	D should make a conscious effort to avoid anger in his/her interactions with others. Perhaps it would be advisable for A to try being happy and friendly and accommodating for short but increasing periods of time when involved in social interactions. Also A could try to take a breath (or count to 25) whenever s/he feels agitated in a social situation.
High score	I don’t think C has to become best friends with his classmate/partner, but he should put forth a little bit of effort to build a rapport. If he is shy too, it could be challenging at first, but if he is not, it might be nice if he took the lead to try and bridge the gap between him and his classmate. Start by discussing the assignment, and if everyone appears comfortable with the conversation, maybe ask what they like to do in their free time, or if they’ve watched any good movies lately. Either way, C will need to speak with their classmate to accomplish the school work, so it might as well be a comfortable and friendly working environment, if attainable.	D needs to sit down with a trusted loved one and explore what is really bothering her. It may be one or two things that is causing her to become so angry. Is it that she’s not getting her way? Is it that she assumes the worst of people? Does she believe others hate her or want to do her harm? Does she have something in her past she’s bitter about? Exploring these issues can help to overcome anger. Then she needs to understand the triggers of her anger and plan what to do specifically when those triggers occur step by step.
***Scale of effectivity***		
**Problem to solve**	A realizes that a friend is avoiding her/him. A wanted very much to make up with him/her.	C is very gullible, which is often exploited by others. Every time s/he was taken advantage of, s/he decided that in the future s/he will be guided by her own interest. Unfortunately, such situations still happen to her/him.
Low score	I would advice A to leave the person they are avoiding even though they are trying to make up with them because it will only produce more anxiety and stress for A. The other person will them when they are ready.	Advice is easy and most of the people done it, but we have to think by our-self what negative we have and when we think about it, we gave valuable word to the nearby hearts.
High score	A needs to reach out to B and get a meeting to discuss what is going on. A needs to realize it might not be the situation she thinks so she keeps an open mind and gets in touch with B. Get a face to face with B and see what is bothering B. If A had anything to do with it apologize and tell B you miss her.	To stop saying ’yes’ right away, but rather to stop, take a step back, and evaluate the situation. He must give himself time to think rather than just react—and if the requester is legitimate they’ll understand this need. If not, that tells him a lot about that person, and they can move away from them right away.

Source: Study results.

### Data analysis

Analyses were performed using IBM SPSS 25 software and RStudio v1.2. The data used in the analyses can be found here: http://cabanski.pl/files/NM_Emotional_and_interpersonal_problem-solving_databases.zip. The assumption of the normal distribution of residuals was examined using Q-Q plots. Whenever there was at least a moderate deviation from linearity, we used a non-parametric test. Otherwise, a parametric test was used. Comparisons between the narrative mindset and control conditions were analyzed using either *t*-test for independent samples or Mann-Whitney *U*. The effect size for *t*-test comparisons was computed using Cohen’s *d*. Correlations were computed using either Pearson’s or Spearman’s coefficients.

The mediation models were assessed using the PROCESS macro for SPSS created by Preacher and Hayes. Specifically, models four and six were used for simple and dual mediation analyses, respectively. The indirect effects were estimated with the bootstrap method of 5000 samples with a 95% confidence interval. The indirect effect is deemed significant if the 95% confidence interval does not cross zero. The estimated regression coefficients were standardized for the continuous variables and partially standardized for the dichotomous variables. The indirect effects were presented in the unstandardized form.

## Results

### Correlations and group comparisons

Across all three scales the narrative mindset group was rated a higher score. The narrative mindset group was judged to have more effective advice (*M* = 3.49, *SD* = .49) than the control group (*M* = 3.19, *SD* = .87), *t*(87.57) = 2.19, *p* = .031, *d* = .42; to be more empathic (*M* = 2.23; *SD* = .79) compared to the control group (*M* = 1.81, *SD* = .64), *t*(107) = 3.10, *p* = .002, *d* = .58; and to be more reliant on personalization of problem (*Mdn* = 1) than the control group (*Mdn* = 1), *Z* = 2.66; *p* = .008. There were no statistically significant differences in *1*^*st*^ *person singular pronouns* (*Z* = 0.13; *p* = .893) and for *past tense verbs* (*Z* = 1.74; *p* = .082). The results are presented in Table 4.

**Table 4:** Comparison of control vs narrative mindset conditions on the effectivity of advice, empathy, personalization of problem and the LIWC measures (the interpersonal problem-solving study).

	Condition					
	Control (*N* = 56)		Narrative (*N* = 53)			
	*M (SD)*	*Mdn*	*M (SD)*	*Mdn*	*p*	
Effectivity of advice	3.19 (.87)	3.38	3.49 (.49)	3.63	.031^t^	
Empathy	1.81 (.64)	1.75	2.23 (.79)	2.00	.002^t^	
Personalization of problem	1.07 (.17)	1.00	1.22 (.33)	1.00	.008^M^	
1^st^ person singular pronouns	0.78 (1.22)	0.00	0.88 (1.56)	0	.893^M^	
Past tense verbs	0.80 (1.06)	0.54	0.45 (0.64)	0	.082^M^	

*Note*: ^t^. *t*-test’s significance; ^M^. Mann-Whitney *U* test’s significance.

The effectivity of advice, empathy, and personalization of problems all correlated positively. Empathy and effectivity of advice were strongly associated (*r* = .66; *p* < .001), while personalization of problem was moderately associated with both effectivity of advice (*rho* = .37; *p* < .001) and empathy (*rho* = .46; *p* < .001).

When tested separately for each of the two conditions, all correlations remained significant and positive, but there was a change in strength in the association between personalization of problem and effectivity of advice. In the narrative mindset condition, the correlation was moderate (*rho* = .45; *p* = .001), while in the control condition it was weak (*rho* = .28; *p* = .036). Empathy correlated strongly with effectivity of advice in both the narrative mindset (*r* = .70; *p* < .001) and control (*r* = .68; *p* < .001) conditions, and the association between personalization of problem and empathy was moderate in both the narrative mindset (*rho* = .47; *p* < .001) and control (*rho* = .38; *p* = .004) conditions.

Spearman’s correlations analyses revealed a positive and weak association between the number of *1*^*st*^ *person singular pronouns* and the problem’s personalization. The correlation occurred for each of the coders’ computed ratings separately as well as the combined rating of the two. No more correlations were found to be significant. The results can be found in Table 5.

**Table 5:** Correlations between LIWC measures and the coders’ assessments on each scale in the interpersonal problem-solving study–Spearman’s coefficient (*N* = 109).

	LIWC	
	*1*^*st*^ *person singular pronouns*	*Past tense verbs*
Personalization of problem		
1^st^ coder	.23*	-.07
2^nd^ coder	.21*	.02
Total	.20*	-.03
Empathy		
1^st^ coder	.11	.13
2^nd^ coder	.08	.03
Total	.09	.09
Effectivity of advice		
1^st^ coder	.11	.07
2^nd^ coder	-.04	.17
Total	.05	.12

* *p* < .05, ** *p* < .01, *** *p* < .001.

### Simple mediation analyses

**Fig 1:**
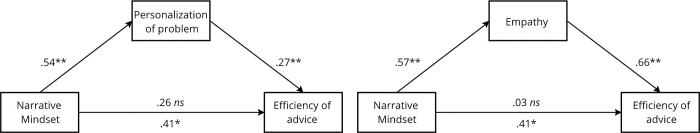
Mediating effect of personalization of problem and empathy on the association between narrative mindset and effectivity of advice–two separate simple mediation models (the interpersonal problem- solving study). *Note*: Presented regression coefficients were partially standardized for the narrative mindset predictor. The remaining were fully standardized. Coding for the independent variable: 0 –control, 1 –experimental. * p < .05. ** p < .01.*** p < .001.

When analyzed using separate simple mediation models, both personalization of problem, BCa CI95% [.04, .19], and empathy, BCa CI95% [.10, .45], mediated significantly the effect of narrative mindset on the effectivity of advice. Each of the models yielded a complete mediation effect, as the direct effects of the narrative mindset on the effectivity of advice were not significant for the model with personalization of problem (*β* = .26, *p* = .171) and empathy (*β* = .03, *p* = .832) respectively. The mediation models are presented in Fig 1.

### Dual-mediation model

**Fig 2:**
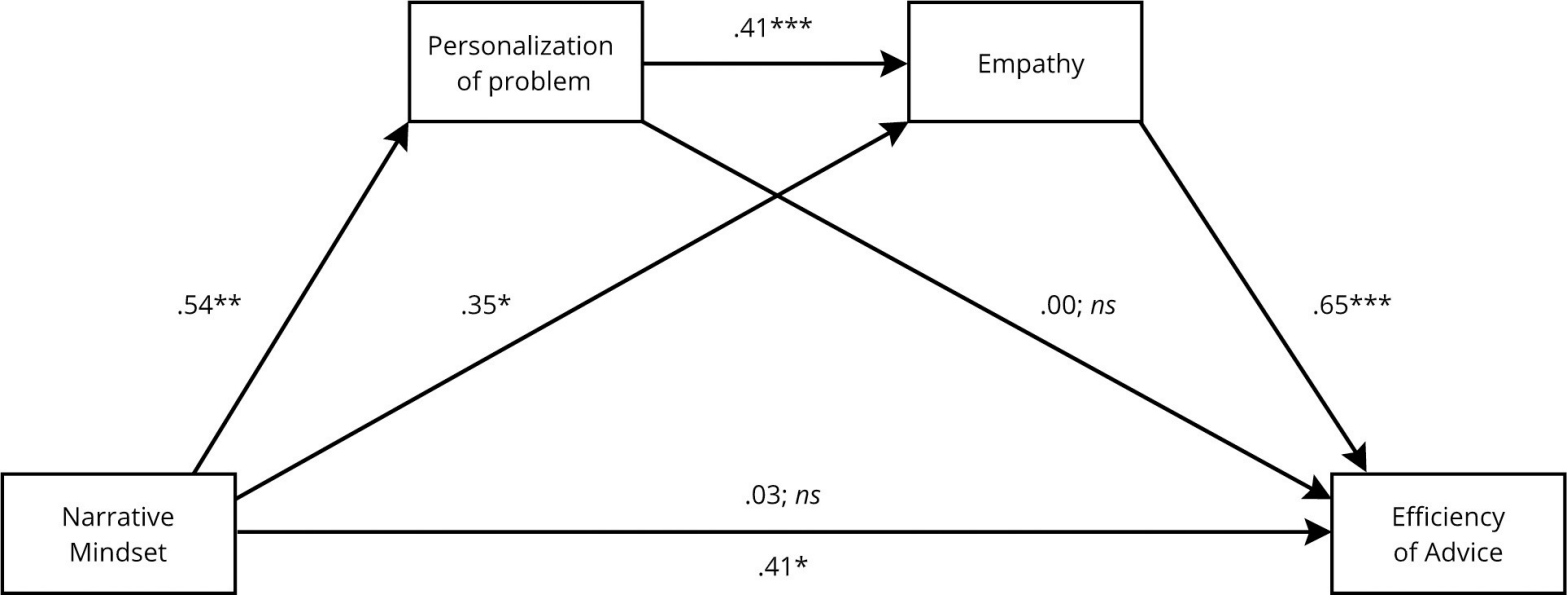
Mediating effect of personalization of problem and empathy on the association between narrative mindset and effectivity of advice–dual-mediation model (the interpersonal problem-solving study). *Note*: Presented regression coefficients were partially standardized for the narrative mindset predictor. The remaining were fully standardized. Coding for the independent variable: 0 –control, 1 –experimental. * p < .05. ** p < .01.*** p < .001.

Inducing a narrative mindset led to a stronger personalization of the problem (*β* = .54, *p* = .004). Both narrative mindset (*β* = .35, *p* = .048) and personalization of problem (*β* = .41, *p* < .001) positively predicted empathy, but there was no indirect effect of narrative mindset on empathy through personalization of problem, BCa CI95% [-.05, .04]. When combined together, empathy positively predicted effectivity of advice (*β* = .65, *p* < .001) while narrative mindset (*β* = .03, *p* = .835) and personalization of problem (*β* = .00, *p* = .995) were not significant. Therefore, there was a complete l mediation effect of the narrative mindset on the effectivity of advice, which was partly mediated through empathy only BCa CI95% [.01, .33] and partly through the dual mediation of personalization of problem and empathy BCa CI95% [.03, .21]. The mediation model and the indirect effects are presented in Fig 2 and Table 6 respectively.

**Table 6:** The indirect effects for the dual-mediation model (the interpersonal problem-solving study).

Indirect effect	*B*	BCa 95% CI
Narrative mindset → Personalization of problem → Effectivity of advice	.00	[-.05, .04]
Narrative mindset → Empathy → Effectivity of advice	.17	[.01, .33]
Narrative mindset → Personalization of problem→ Empathy → Effectivity of advice	.11	[.03, .21]
Total	.28	[.09, .46]

The 95% confidence intervals were estimated based on 5000 bootstraps.

## Experiment 2: Emotional problem-solving

### Participants

This experiment had the same structure, rules of participant recruitment, and incentive as experiment 1. In total, 120 mTurk workers (53 men and 67 women) participated in the study. Independent coders decided that data from 13 participants (8 from the narrative condition) cannot be used because of one or two reasons: (a) possible cheating (b) providing text that was irrelevant to the task, possibly copied from the Internet. Finally, there were 56 participants in the Narrative mindset and 64 in the Control condition. Detailed demographics can be found in Table 7.

**Table 7:** Demographics of the participants in the emotional problem-solving study.

	*n*	*%*	*M*	*SD*
Age				
Total sample	120		40.53	10.10
Female	67	55.8%	40.09	10.30
Male	53	44.2%	41.09	9.92

### Procedure and materials

After the mind-setting part, participants were invited to the second part of the experiment: the emotional problem task. They were asked to provide advice for four people dealing with particular states with a high emotional component. Problem descriptions were inspired by the works of Appel and Kaestner [2] and Siegel and others [5]. They were:

"A dreams about a career in acting, but worries, because during public speeches, A is always very anxious about others and feels embarrassed.

The husband/wife of B died five years ago in a car accident. Although a lot of time has passed, B is depressed. Almost nothing can put him/her in a good mood, s/he does not see much sense in anything s/he does. B would like to feel like s/he used to.

C is very gullible, which is often exploited by others. Every time s/he was taken advantage of, s/he decided that in the future s/he will be guided by her own interest. Unfortunately, such situations still happen to her/him.

D often explodes with anger and becomes aggressive towards others, even towards loved ones. S/he has the impression that people are starting to avoid him/her. D feels that s/he cannot control his/her bad emotions."

### Data analysis and advice evaluation

Coders’ assessment and LIWC analyses were performed in the same manner as in experiment 1. Each pair of coders worked on the same scale in both experiments. The same statistical analyses, as in experiment 1, were used.

Evaluations provided by coders correlated significantly. Effectivity scale *r* = .94 (*p* < .001); empathy scale, *r* = .89 (*p* < .001) and personalization scale *r* = .93 (*p* < .001).

Like in experiment 1, the range of the correlations is similar to correlation in other studies using this measure. Example pieces of advice are presented in Table 3.

The interrater reliability was found to be satisfactory–the level of agreement between the coders was from moderate to substantial. Table 8 presents the computed weighted kappa coefficients.

**Table 8:** Interrater reliability of the coders’ evaluations of the pieces of advice—linearly weighted kappa (the emotional problem-solving study).

Advice	Effectivity of advice	Empathy	Personalization of problem
1	.78	.58	.69
2	.73	.61	.70
3	.63	.54	.59
4	.69	.51	.65

## Results

### Correlations and group comparisons

Similarly to experiment 1, the narrative mindset group was rated higher than the control group across all three scales. The narrative mindset group was judged to be more effective (*M* = 3.51; *SD* = .81) than the control group (*M* = 3.20; *SD* = .64), *t*(118) = 2.40; *p* = .018; *d* = .42; more empathic (*Mdn* = 1.88) compared to the control group (*Mdn* = 1.50), *Z* = 4.21; *p* < .001; and more reliant on personalization of problem (*Mdn* = 1.63) than the control group (*Mdn* = 1.13), *Z* = 3.12; *p* = .002. Additionally, the narrative mindset group (Mdn = 0.90) used significantly more *1*^*st*^ *person singular pronouns* in their advices than the control group (Mdn = 0.00), *Z* = 2.49; *p* = .013. There were no significant differences in the use of *past tense verbs*, *Z* = 1.48; *p* = .139. The results are presented in Table 9.

**Table 9:** Comparison of control vs. narrative mindset conditions on the effectivity of advice, empathy, personalization of problem, and the LIWC measures (the emotional problem-solving study).

	Condition				
	Control (*N* = 64)		Narrative (*N* = 56)		
	*M (SD)*	*Mdn*	*M (SD)*	*Mdn*	*p*
Effectivity of advice	3.20 (.64)	3.28	3.51 (.81)	3.65	.018^t^
Empathy	1.59 (.51)	1.50	2.14 (.82)	1.88	< .001^M^
Personalization of problem	1.38 (.49)	1.13	1.75 (.74)	1.63	.002^M^
1^st^ person singular pronouns	0.99 (1.46)	0.00	2.10 (2.46)	0.90	.013^M^
Past tense verbs	0.76 (1.11)	0.22	1.00 (1.22)	0.84	.139^M^

*Note*: ^t^. *t*-test’s significance; ^M^. Mann-Whitney *U* test.

The effectivity of advice, empathy, and personalization of problems, like in experiment 1, all correlated positively. This time, a strong association was between empathy and personalization of problem (*rho* = .64; *p* = < .001), while effectivity of advice was moderately associated with empathy (*rho* = .43; *p* < .001) and personalization of problem (*rho* = .35; *p* < .001).

However, there was a change in correlations when they were tested independently for each of the two conditions. In the control condition, effectivity of advice ceased to correlate with empathy (*rho* = .16; *p* = .219) and personalization of problem (*rho* = .23; *p* = .067), which is in contrast to the narrative mindset condition, where it correlated strongly with empathy (*rho* = .53; *p* < .001) and moderately with personalization of problem (*rho* = .37; *p* = .005). The association between empathy and personalization of problem remained strong in both the narrative mindset (*rho* = .63; *p* < .001) and control condition (*rho* = .55; *p* < .001).

The number of *past tense verbs* was positively correlated with the personalization of problem and empathy. The associations were mostly moderate and occurred for each of the coders’ ratings separately and for the combined evaluations. The number of *1*^*st*^ *person singular pronouns* was positively and weakly associated with personalization of problem and effectivity of advice (for each of the three evaluations) and the 1^st^ coder’s evaluation of empathy. The results can be found in Table 10.

**Table 10:** Correlations between LIWC measures and the coders’ assessments on each scale in the emotional problem-solving study–Spearman’s coefficient (*N* = 120).

	LIWC	
	*1*^*st*^ *person singular pronouns*	*Past tense verbs*
Personalization of problem		
1^st^ coder	.22*	.41***
2^nd^ coder	.21*	.36***
Total	.21*	.40***
Empathy		
1^st^ coder	.19*	.36***
2^nd^ coder	.13	.28**
Total	.16	.34***
Effectivity of advice		
1^st^ coder	.21*	.10
2^nd^ coder	.24**	.10
Total	.24**	.12

* *p* < .05

** *p* < .01

*** *p* < .001.

### Simple mediation analyses

**Fig 3:**
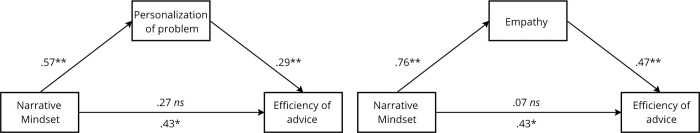
Mediating effect of personalization of problem and empathy on the association between narrative mindset and effectivity of advice–two separate simple mediation models (the emotional problem-solving study). *Note*: Presented regression coefficients were partially standardized for the narrative mindset predictor. The remaining were fully standardized. Coding for the independent variable: 0 –control, 1 –experimental. * p < .05. ** p < .01.*** p < .001.

When analyzed using separate simple mediation models, both personalization of problem, BCa CI95% [.02, .25], and empathy, BCa CI95% [.14, .41], mediated significantly the effect of narrative mindset on the effectivity of advice. Each of the models yielded a complete mediation effect, as the direct effects of the narrative mindset on the effectivity of advice were not significant for the model with personalization of problem (*β* = .27, *p* = .145) and empathy (*β* = .07, *p* = .682) respectively. The mediation models are presented in Fig 3.

### Dual-mediation model

**Fig 4:**
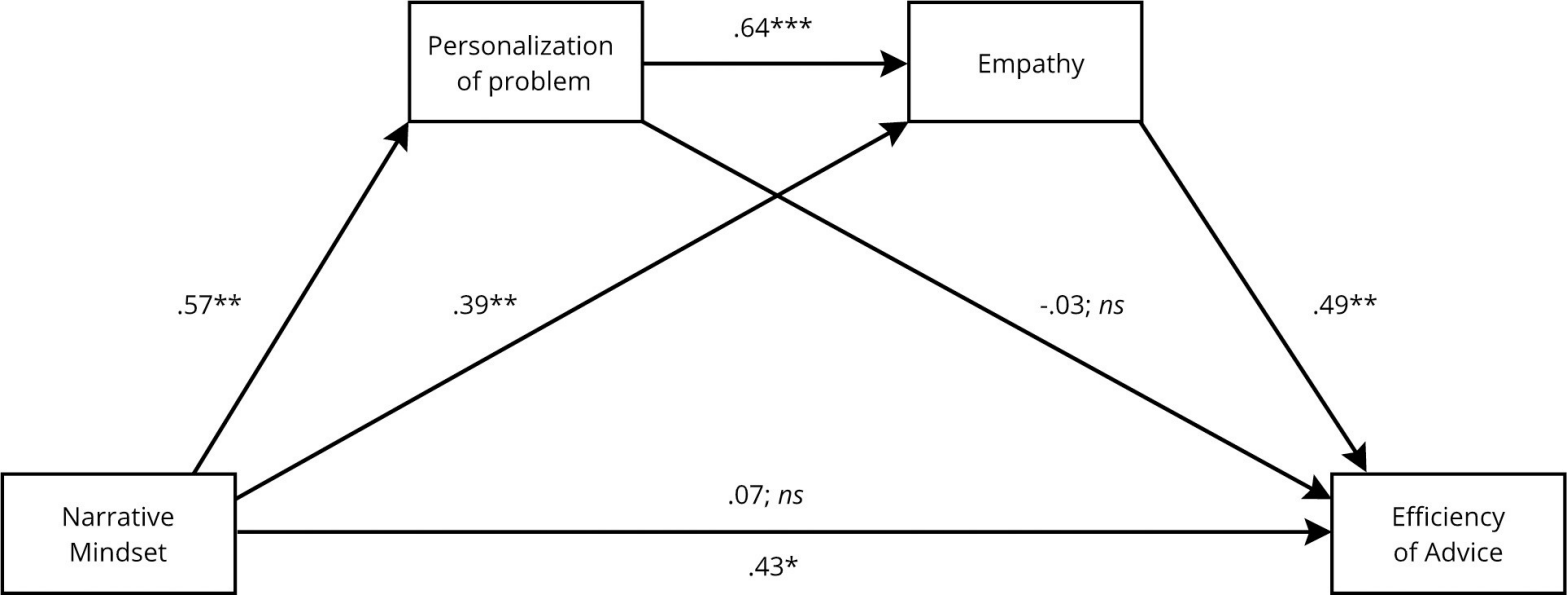
Mediating effect of personalization of problem and empathy on the association between narrative mindset and effectivity of advice–dual-mediation model (the emotional problem-solving study). *Note*: Presented regression coefficients were partially standardized for the narrative mindset predictor. The remaining were fully standardized. Coding for the independent variable: 0 –control, 1 –experimental. * p < .05. ** p < .01.*** p < .001.

Inducing a narrative mindset led to a higher personalization of problem (*β* = .57, *p* = .002). Both narrative mindset (*β* = .39, *p* = .004) and personalization of problem (*β* = .64, *p* < .001) positively predicted empathy and there was no indirect effect of narrative mindset on empathy through personalization of problem, BCa CI95% [-.13, .10]. When combined together, empathy positively predicted effectivity of advice (*β* = .49, *p* < .001) while narrative mindset (*β* = .07, *p* = .677) and personalization of problem (*β* = -.03, *p* = .788) were not significant. This means that the effect of the narrative mindset on the effectivity of advice was fully mediated—partly by the indirect effect through empathy only BCa CI95% [.05, .27] and partly by the indirect effect through personalization of problem and then empathy BCa CI95% [.04, .26]. The mediation model and the indirect effects are presented in Fig 4 and Table 11 respectively.

**Table 11:** The indirect effects for the dual-mediation model (the emotional problem-solving study).

Indirect effect	*B*	BCa 95% CI
Narrative mindset → Personalization of problem → Effectivity of advice	-.01	[-.13, .10]
Narrative mindset → Empathy → Effectivity of advice	.14	[.05, .27]
Narrative mindset → Personalization of problem→ Empathy → Effectivity of advice	.14	[.04, .26]
Total	.26	[.13, .42]

The 95% confidence intervals were estimated based on 5000 bootstraps.

## Summary of the results

Analyses revealed that the narrative mindset, regardless of the type of a problem, led to higher empathy and personalization of problem, and finally to the higher effectivity of advice, thus supporting our basic hypotheses. Simple mediation analyses showed that, regardless of the type of problem, both personalization of problem and empathy–on their own–mediated completely the effect of narrative mindset on the effectivity of advice. When analyzed together in a dual-mediation model, the effect of narrative mindset on the effectivity of advice was partly mediated through empathy only and partly through personalization of problem and empathy combined, while the isolated mediation effect of personalization of problem ceased to be significant. No direct effect of the narrative mindset on the effectivity of advice was found. Overall, in both emotional and interpersonal situations, the narrative mindset simultaneously induces empathy and personalization of problems and finally increases problem-solving.

Additionally, we observed some interesting differences in the pattern of correlations between the two studies. In the case of emotional problems, the positive influence of empathy and personalization on advice’s effectivity was observed only in the narrative mindset, while in the case of interpersonal problems, the correlation between personalization and effectivity of advice was stronger in the narrative mindset than in the control condition. Further, the association between personalization of a problem and empathy was stronger in the emotional problem study. Also, in the emotional problem study, the analysis revealed more effects for the LIWC measures, among which were a higher proportion of *1*^*st*^ *person singular pronouns* in the narrative condition and the correlations of personalization of problem and empathy with both *1*^*st*^ *person singular pronouns* and *past tense verbs*.

In general, the results seem to support our hypothesis that in the narrative mindset increased empathy, supported by the co-occurring increase in the personalization of the problem, leads to higher effectivity of the advice.

## Discussion

In our exploration, we were interested in specific, situationally invoked mental processes–the narrative mindset–that influence interpretation and coping with interpersonal or emotional problems. The results suggest that in interaction with another person’s problem, the narrative mindset enables people to understand the problem better and find more effective advice. Better empathy and personalization of the problem appear to be the main, although probably not the only, reason for this positive effect. Following these findings, the correlation between personalization of the problem and the effectivity of advice were higher in the narrative mindset than in the control condition. Moreover, it was observed primarily in emotional problems where there was no significant correlation between personalization and effectivity in the control condition. This higher personalization of other person’s problems in the narrative mindset was indicated not only in the coders’ assessment but also in more objective LIWC analyses of frequencies of words related to personal experience, that is, words belonging to the linguistic categories *1*^*st*^ *person singular pronouns* and *past tense verbs* [94]. As expected, words from the *1*^*st*^ *person singular pronouns* category appeared more frequently in the advice invented in the narrative mindset in case of emotional problems [5].

Additionally, correlational analyses indicated that in the case of emotional problems, the positive influence of empathy and personalization on advice’s effectivity was observed only in the narrative mindset. Also, in the case of interpersonal problems, the correlation between personalization and advice effectivity was more robust in the narrative mindset than in the control condition. These findings may suggest that the narrative mindset increases perspective taking of another person in need and makes empathy and personalization more productive in helping others.

The presented studies have several imitations. First of all, there was no direct measure of the role of episodic memories and other kinds of experiential knowledge engaged in solving another’s problem. The measurement was based mainly on coders’ work, which subjectively evaluated the role of personal experiences and empathy revealed in the content of advice. Supporting data, however still indirect, came from the LIWC [93].

Further experiments should allow the direct observation of these relationships. One possibility is to ask participants, immediately after the advice presentation, to describe their thought process when considering the problem and solution. Observations would include the frequency and elaboration of the content related to personal experiences. Another solution is to ask a participant, immediately after the problem-solving task, to describe their own (or loved ones’) experiences similar to those experienced by the target person. Observations would include the frequency and elaboration of this content. The collected material from those two kinds of observations would make it possible to test the expectation that the narrative mindset enables activation of specific personal experiences within memory and makes them more elaborate else’s problems. It would also allow us to investigate further the particular role and moment of personalization in understanding and to solve such problems.

In future studies, we should consider that applying our own experiences in advising another person does not necessarily result in positive outcomes. First, merely redirecting attention from another person’s difficulties to personal memories may disturb empathizing and understanding of the other person’s problems. It may be a way to escape from their difficulties and the effort needed to help [102–104]. An example from our study is advice for a person struggling after the death of a spouse. One participant wrote only: "Not sure, my dad is like this after the death of my mom, and that was five years ago". Secondly, applying their personal experiences to solve someone else’s problem may limit the spectrum of searching for a specific solution in that person’s case. In Piagetian terms, it may block decentration.

The literature on the narrative mindset’s impact is relatively rare besides recognition of the crucial role of narratives in culture and human ways of remembering, reasoning, and behaving. The presented results do not yet lead to an empirically supported and elaborated model of the narrative mindset’s impact on social problem-solving and social cognition in general. However, we hope that they provide a background for the hypotheses and help open new inquiries. Below are topics that are worth elaboration and may lead to further research.

Understanding another person’s difficulties requires time and engagement in changing perspectives to see the situation from their point of view. It might be costly. This work involves motivation and cognitive effort [105, 106]. Furthermore, there are also emotional costs of empathizing with other people with troubles [103]. Additionally, concerns about possible empathetic errors make empathy feel effortful [105, 107]. The narrative mindset probably makes such engaging and thinking more fluent and effortless. We better understand others people’s situations and their way of experiencing them, and eventually, we may find an adequate solution. Additionally, this cognitive fluency and effectivity may lower emotional costs because a person is more self-confident and more concerned about the other person’s problem than their emotions and self-evaluation.

We may suppose that two processes are facilitated by the narrative mindset, perspective taking and applying personal experiences to understand others, may contribute to breaking social stereotypes and prejudice. We observed it in our earlier studies [62]. Within the narrative mindset, another person should become less alien to us and understood not merely as a member of some social category or group but also as a person who has feelings, individual motives, experiences, and life stories. It helps to understand and advise this person. There are possible other correlated benefits. Psychological distance to this person becomes shortened–a feeling that they are more like me. It may reduce negative attributions and dehumanized person impressions [108]. Moreover, in the narrative mindset, a person’s image should be more distinct and elaborated cognitively, and a person gets our attention. As a result, our moral values and social standards would be easier to apply when interacting with this person [109, 110].

Supposing that the narrative mindset facilitates episodic memory use in dealing with a problem, it could be helpful as a part of the therapy of difficulties connected with the disordered processing of episodic memories. They include depression, anxiety, stress-related disorders [111], natural aging [112] and children with autism spectrum disorder [113]. Only clinical studies may elaborate on this point and test the hypothesis.

In the end, we think that one easy to apply implication of this study for everyone helping others may be: if you want to help a person, at first try to understand misfortune within their self-story frame, not in a handbook on problem-solving.
